# Evaluation of erythroblast macrophage protein related to erythroblastic islands in patients with hematopoietic stem cell transplantation

**DOI:** 10.1186/2047-783X-18-9

**Published:** 2013-04-08

**Authors:** Xiaolu Mao, Xiaoyan Shi, Feng Liu, Guining Li, Lihua Hu

**Affiliations:** 1 Department of Clinical Laboratory, The Central Hospital of Wuhan, Wuhan, 430014, China; 2 Department of Central Laboratory, The Central Hospital of Wuhan, Wuhan, 430014, China; 3 Department of Blood Transfusion, Union Hospital, Tongji Medical College, Huazhong University of Science and Technology, Wuhan, 430014, China

**Keywords:** Erythroblast macrophage protein, Cancer, Hematopoietic stem cell transplantation, Hematopoiesis, Erythroblastic island

## Abstract

**Background:**

Hematopoietic evaluation of the patients after Hematopoietic stem cell transplantation (HSCT) is very important. Erythroblast macrophage protein (Emp) is a key protein with function in normal differentiation of erythroid cells and macrophages. Emp expression correlates with erythroblastic island formation, a process widely believed to be associated with hematopoiesis in bone marrow. We aimed to investigate the hematopoietic function of bone marrow from 46 HSCT patients and 16 inpatients with severe anemia applied to the treatment of EPO by measuring Emp expression level.

**Methods:**

Emp mRNA and protein expression levels in mononuclear cells of bone marrow and peripheral blood samples were detected by RT-PCR and Western blotting method respectively.

**Results:**

While hematopoiesis occurs in bone marrow, Emp expression level was elevated and more erythroblastic islands were found , and Emp is upregulated in bone marrow in response to erythropoietin (EPO) treatment.

**Conclusions:**

Emp expression correlates with erythroblastic island formation and has an important function for bone marrow hematopoiesis. Emp could be a potential biomarker for hematopoietic evaluation of HSCT patients.

## Background

The erythroblastic island is a distinct anatomic unit which consists of a central macrophage surrounded by erythroid cells. These islands are present at various stages of differentiation in the fetal liver and the adult bone marrow, and have a key role in erythroid cell proliferation and differentiation [1-4]. Gregory and Eaves found that the erythroid cells differentiated through morphologically defined stages of erythroid progenitors, proerythroblasts, basophilic erythroblasts, polychromatophilic erythroblasts, and orthochromatophilic erythroblasts [5,6]. The orthochromatophilic erythroblasts then underwent enucleation by extruding their nucleus and became reticulocytes, further expelling all organelles and detaching from their microenvironment to form mature circulating erythrocytes. Over the course of erythroid differentiation, erythroblasts displayed a gradual decrease in cell size and an increase in hemoglobin concentration [7]. The major changes of erythroid cells occurred in erythroblastic island, which correlate with the hematopoiesis of bone marrow. Despite recent advances, the mechanism of erythroblast maturation remains poorly understood. Previous reports showed that within erythroblastic islands, both erythroblast-macrophage and erythroblast-erythroblast maturation occurred by extensive interactions [1,2,8-12]. Interestingly, only a few molecules have been definitively shown to enhance the formation of erythroblastic islands. The adhesive interaction involved α4β1 integrin and vascular cell adhesion molecule-1 (VCAM-1) were first identified in erythroblasts and macrophages, respectively [13]. Recently, the role of ICAM-4 in erythroblastic island formation has been clearly documented by gene targeting *in-vivo* studies [14,15]. Emp was initially identified as a mediator of erythroblast-macrophage interactions during erythroid differentiation [1,2,8,9]. More recently, it was thought that Emp had an important role in erythroblastic island formation [8,16,17]. Using a transplant combination from Emp null fetal liver cells to lethally irradiated wild-type sibling mice, Soni*et al.* found that loss of Emp function in erythroid cells resulted in impaired proliferation and terminal differentiation[18]. These findings suggest that Emp protein might be a potential molecular marker for hematopoietic evaluation.

Hematopoietic stem cell transplantation (HSCT) is the transplantation of stem cells derived from the bone marrow or blood [19]. Stem cell transplantation is a medical procedure used in the fields of hematology and oncology. Increase in the number of erythroblastic islands in bone marrow always suggests a successful HSCT. However, no clinical correlation between Emp levels and hematopoiesis has been established. In this study, we evaluated hematopoietic function of bone marrow in HSCT patients through the change of Emp expression before and after HSCT. Based on our data, we suggest that Emp is a potential biomarker for hematopoietic evaluation of HSCT patients.

## Methods

### Patient characteristics of HSCT

The use of human cord blood and peripheral blood was approved by Medical Ethical Committee of the Tongji Medical College. Approval was granted in accordance with Chinese legislations, and written informed consent was obtained from all participants, in accordance with the guidelines of the Chinese Ministry of Health. HSCT recipients at the HSCT program of the Hematology and Hemotherapy Discipline at Tongji Medical College, Huazhong University of Science and Technology were prospectively enrolled in this trial. Forty-six patients, HSCT recipients from September 2008 to December 2009, were eligible for the study. Thirty-one patients underwent allogeneic HSCTs, and 15 patients received autologous HSCTs. In the allogeneic HSCT group, all patients received HSCTs from a human leukocyte antigen (HLA) of identical donor, and no patient received a T-cell depleted HSCT. Demographic details for all 46 patients are shown in Table 1.

**Table 1:** Patient characteristics, HSCT type, underlying disease, and immunosuppressive regimens used before HSCT

**Gender**		***n *(%)**
	Female	20 (43.5)
	Male	26 (56.5)
Age		Years (range)
	Mean	32 (18–37)
HSCT		*n *(%)
	Allogeneic	31 (67.4)
	Autologous	15 (32.6)
Underlying disease		*n *(%)
	Chronic myelogenous leukemia	26 (56.5)
	Acute myelogenous leukemia	8 (17.4)
	Multiple myeloma	6 (13.1)
	Hodgkin’s lymphoma	3 (6.5)
	Severe aplastic anemia	3 (6.5)
Immunosuppressive conditioning regimen		*n *(%)
	Busulfan and melphalan	30 (65.2)
	Busulfan and cyclophosphamide	4 (8.7)
	Total body irradiation and melphalan	3 (6.5)
	Total body irradiation and cyclophosphamide	3 (6.5)
	Other	6 (13.1)

Peripheral blood and bone marrow were taken from HSCT recipients at the point of 30, 90, and 180 days, respectively. Before HSCT, all patient samples of peripheral blood and bone marrow were acted as the second control group, and that 10 donors constituted the first control group. Samples were processed for peripheral blood and bone marrow smears, and isolation of mononuclear cells.

### Isolation of mononuclear cells in peripheral blood and bone marrow

Heparinized (10 IU/mL) peripheral blood and bone marrow was layered on a Ficoll-Hypaque discontinuous gradient system (Sigma) and centrifuged at 1200 × g for 30 min. The mononuclear cells at the interface of plasma and Ficoll-Hypaque were collected and re-suspended in serum-free dulbecco’s modified eagle medium (DMEM). Then the quantity (A) of mononuclear cells were detected by the blood cell analyzer (Beckman Coulter), and their smears were examined by Wright/Giemsa staining (Maker Biotechnology Co., China) for the erythroblastic cell ratio (B%) of mononuclear cells. The erythroblastic cell of number was calculated using the equation: erythroblastic cells (× 10^9^/L) = A(× 10^9^/L) × B%

### Morphology

The morphology of peripheral blood and bone marrow cells was examined by Wright/Giemsa staining. In addition to using blood cell analyzer, cells were directly smeared onto glass plate in an appropriate cell concentration (10 μL blood or bone marrow) and stained with Wright/Giemsa solution. The morphology of blood cells was observed under microsope and at least 200 blood cells with nucleus were counted to determine the ratio of erythroblastic cell in peripheral blood, or the hyperplastic degree in bone marrow.

### Semi-quantitative RT-PCR

Mononuclear cells in bone marrow and peripheral blood samples were adjusted to the same quantity (1 × 10^6^ cells in PBS). Total RNA was extracted from the cells and single strand cDNA synthesis was performed by using Blood RNA extraction kit (QIAGEN, Hilden, Germany) and moloney murine leukemia virus-reverse transcription kit (SuperSciptII, Life Technologies, Inc.) according to the manufacturer’s directions. PCR primers used for Emp and β-actin mRNA detection are as follows: Emp sense 5’-ACCCGACCCTCAAGGTGCCC-3’, antisense 5’-GTGGCCG TCTCACGCCTCTC-3’; β-actin5’-TTCCTGGGCATGGAGTCCT-3’, antisense 5’-TGATCTTCATTGTGCTGGGTG-3’. The gene expression levels were normalized by β-actin.

### Western blot

Cells were lysed with lysis buffer (20 mMTris–HCl pH 7.8, 1 mM EDTA, 50 mM sodium chloride, and 0.5% NP-40), and protein concentration was determined by BCA assay kit (Pierce, USA). Western blot analysis was carried out as directions of manufacturer (Cell signaling Biotech). Antibodies against human Emp (Abcam plc. USA. ab65239) and β-actin (Cell signaling Biotech, USA) were used. Results were visualized with horseradish peroxidase-conjugated secondary antibodies (Sigma, USA; 1:1000) and enhanced chemiluminescence.

### EPO treatment

Use of erythropoietin (EPO) hormone as an anti-apoptotic permitted survival of erythroid progenitors from erythrocyte colony-forming unit (CFU-E) through early proerythroblast stages of differentiation [20]. A total of 16 inpatients with severe anemia were applied to the treatment of EPO (rhu EPO, 75 u per kg, 3 times weekly, subcutaneous). Pre- and post-EPO treatment bone marrow samples were obtained for morphology and Emp expression analysis. There were six women and 10 men among 16 inpatients, and their mean age was 33 years. The mean hemoglobin level in 16 inpatients was 46.3 ± 2.3 g/L before EPO treatment. Erythroblastic islands were not visible in their bone marrow. The inpatients characteristics before EPO treatment are shown in Table 2.

**Table 2:** The patient characteristics before EPO treatment

**Gender**		***n *(%)**
	Female	6 (37.5)
	Male	10 (62.5)
Age		Years(range)
	Mean	33 (24–37)
Hb		g/L
	Mean	46.3 ± 2.3
Bone marrow		Mean value in 12 inpatients with severe anemia
	BMHD	Low or extremely low
	MMCs	4.7 ± 1.2 × 10^9^/L
	MERm	15.4 ± 1.3%
	Island	Invisibility
	Emp protein	The Emp strip was invisible

BMHD, Bone marrow hyperplastic degree; Hb,Hemoglobin; MERm, Mean erythroblastic cells of ratio in mononuclear cells; MMCs,mean mononuclear cells.

### Statistical analysis

Statistical analysis between groups was performed using Student t-test. Results were considered statistically significant if *P*values were <0.05.

## Results

### Analysis of morphology

**Figure 1:**
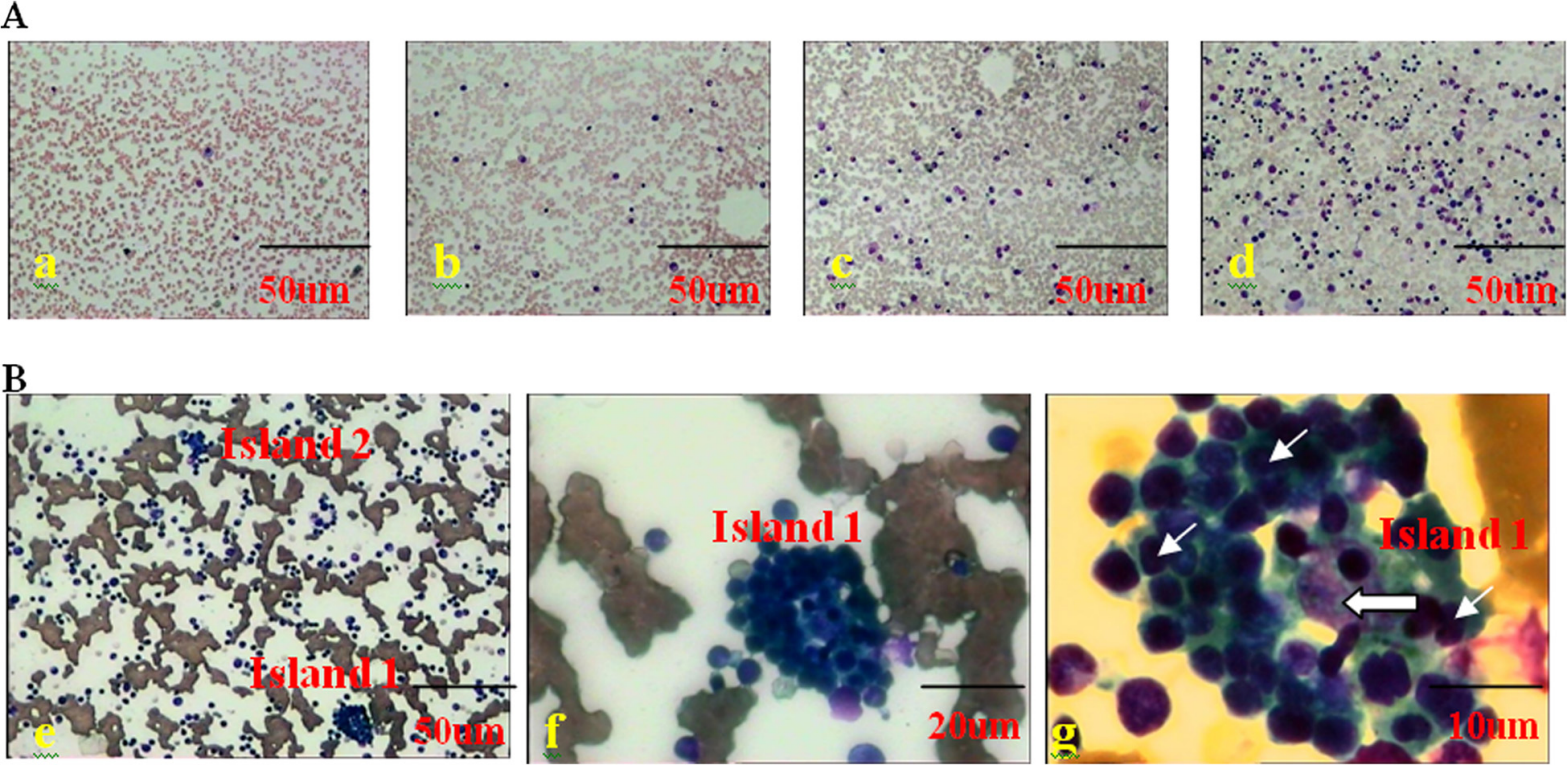
**The hyperplastic degree and morphology of erythroblastic islands in bone marrow.** (**A**) The bone marrow hyperplastic degree. Extremely low (a), low (b), active (c), more active (d). The images were taken under 100 × magnification. (**B**) Morphology of bone marrow erythroblastic islands was shown under 100× (e), 400× (f), and 1,000 × magnification (g). The larger arrow pointed to the macrophage and the smaller ones indicated erythroblasts.

To analyze the hematopoietic function of bone marrow in different groups, morphology analysis was conducted in all samples.In the bone marrow donor group, the MWBCs (Mean White Blood Cells) is 5.7 ± 1.3 × 10^9^/L and the MMCs (Mean Mononuclear Cells) is 2.7 ± 1.5 × 10^9^/L in peripheral blood. The MWBCs and MMCs in peripheral blood from inpatients pre- and post-HSCT were lower than that in the peripheral blood from bone marrow donor group. Whereas, the MERw (the percentage of erythroblastic cells in white blood cells) and the MERm (the percentage of erythroblastic cells in mononuclear cells) were lower in peripheral blood from the bone marrow donor group than that in peripheral blood from inpatients pre- and post-HSCT (Table 3). The morphological characteristics of bone marrow were described by the bone marrow hyperplastic degree (BMHD) and number of erythroblastic islands. The bone marrow hyperplastic activity was examined in the donor group, pre-HSCT, and post-HSCT groups. The BMHD was low or extremely low in the pre-HSCT group. Ninety days after HSCT, it showed an elevation in hematopoietic activity (Table 3, Figure 1A). The MIQASs (Mean Islands Quantity In a Smear) of bone marrow erythroblastic islands (Table 3, Figure 1B) in the groups were as follows: the donor group (21.4 ± 4.5), the pre-HSCT group (3.2 ± 1.4), 30 days after HSCT (43.3 ± 4.1), 90 days after HSCT (46.2 ± 3.6), and 180 days after HSCT (44.7 ± 4.2). The MIQAS in the donor group was higher than that in the pre-HSCT group, but was less than that in post-HSCT groups. However, The MMCs and MERm in the bone marrow from bone marrow donor group were higher than that in the bone marrow from the pre- and post-HSCT groups (Table 3).

**Table 3:** Characteristics of morphology before or after HSCT

**Groups**	**Peripheral blood**				**Bone marrow**			
	**MWBCs (×10**^**9**^**/L)**	**MERw (%)**	**MMCs (×10**^**9**^**/L)**	**MERm (%)**	**BMHD**	**MIQAS**	**MMCs (×10**^**9**^**/L)**	**MERm (%)**
The first control	5.7 ± 1.3	1.1 ± 0.4	2.7 ± 1.5	1.4 ± 0.5	Activity	21.4 ± 4.5	12.8 ± 2.5	48 ± 4.3
The second control	2.1 ± 0.7	6 ± 1.2	1.7 ± 0.6	10.3 ± 1.7	Low or extremely low	3.2 ± 1.4^a^	5.3 ± 1.4^a^	16.7 ± 1.6^a^
30 days after HSCT	2.5 ± 1.0^a^	13.6 ± 1.6^a^	1.5 ± 0.4	17.8 ± 1.9^a^	Activity	43.3 ± 4.1^a,b^	6.6 ± 1.7^a^	23.5 ± 1.2^a^
90 days after HSCT	3.2 ± 1.2^a^	15.4 ± 1.8^a^	2.2 ± 0.7	19.7 ± 1.4^a^	More activity	46.2 ± 3.6^a,b^	10.4 ± 1.5^a^	31.2 ± 1.8^a^
180 days after HSCT	3.0 ± 1.1^a^	14.2 ± 1.5^a^	2.1 ± 0.8	18.5 ± 1.6^a^	Activity	44.7 ± 4.2^a,b^	8.8 ± 1.2^a^	33.4 ± 1.1^a^

^a^*P* < 0.05, *vs.* the first control group.

^b^*P* < 0.05, *vs.* the second control group.

BMHD, Bone marrow hyperplastic degree; MERm, mean erythroblastic cells of ratio in mononuclear cells; MERw, Mean erythroblastic cells of ratio in white blood cells; MIQAS, Mean islands quantity in a smear; MMCs, mean mononuclear cells; MWBCs,Mean white blood cells.

### The Emp expression

**Figure 2:**
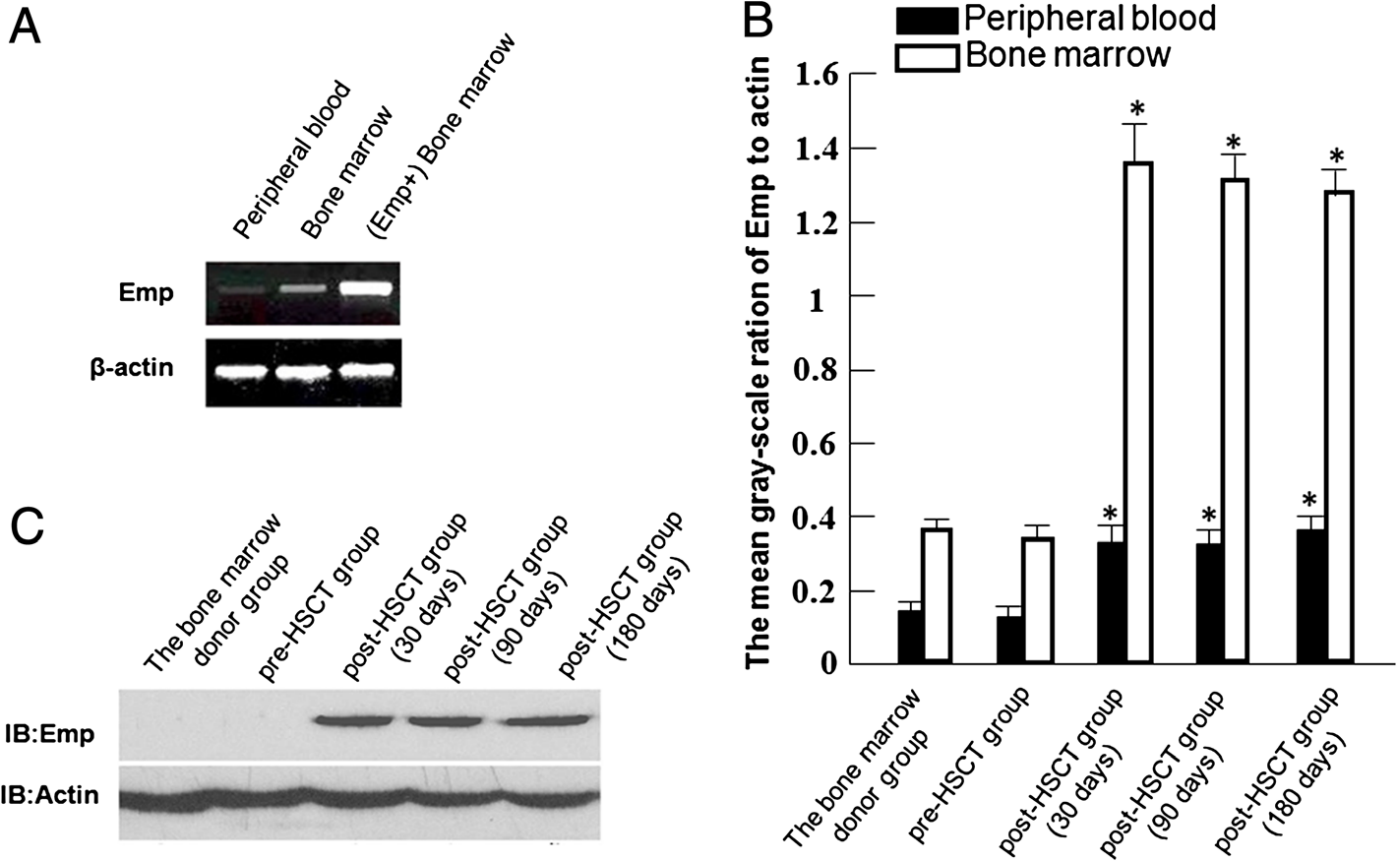
**Emp expression levels in peripheral blood and bone marrow.** (**A**)Emp protein levels in bone marrow in pre- and post-HSCT groups. (**B**)Emp mRNA levels in peripheral blood, bone marrow and Emp protein-positive bone marrow. (**C**) The mean gray-scale ratio of Emp to β-actin in peripheral blood and bone marrow from the bone marrow donor group, pre-HSCT group and day 30, 90, or 180 post-HSCT groups. These data are representative of three independent experiments. *n* = 3. *P < 0.05.

Emp protein and mRNA expressions were detected in mononuclear cells from peripheral blood and bone marrow. Emp protein was not detected in peripheral blood pre-HSCT and post-HSCT groups. Emp protein was undetectable in bone marrow in pre-HSCT group, but was detected in post-HSCT group, and there is no difference between day 30, 90, and 180 post- HSCT groups (Figure 2A). Emp mRNA level was higher in Emp protein-positive bone marrow than that in Emp protein-negative bone marrow (*P* < 0.05) (Figure 2B). Furthermore, Emp mRNA levels in mononuclear cells from peripheral blood and bone marrow were significantly increased after HSCT (*P* < 0.05) (Figure 2C).

### Analysis of EPO treatment

**Figure 3:**
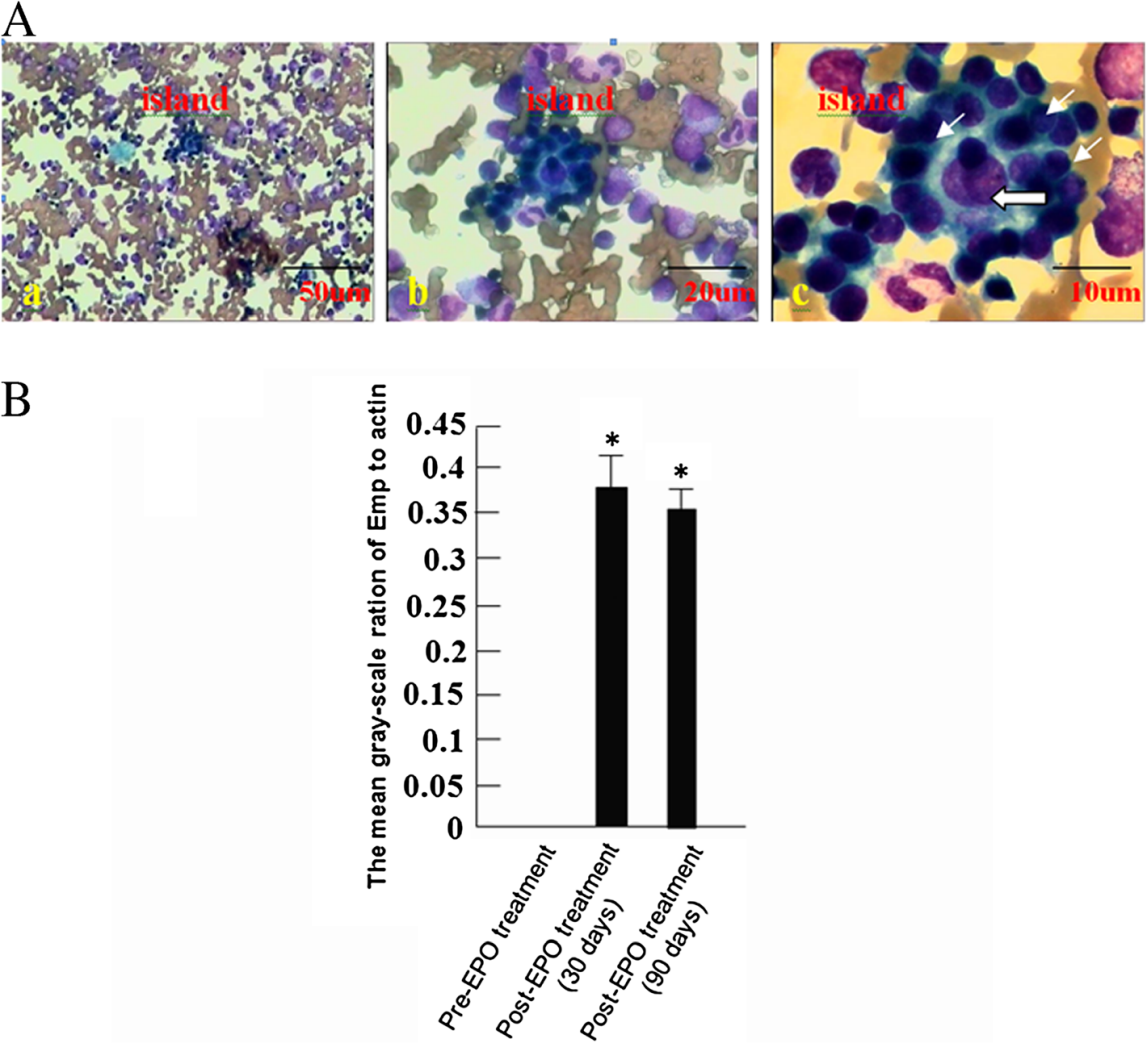
**Emp expression after EPO treatment in bone marrow.** (**A**). The bone marrow island in one of 16 patients treated with EPO was shown under 100× (a), 400× (b), and 1,000 × (c) magnification. The larger arrow pointed to the macrophage and the smaller ones indicated erythroblasts. (**B**) The mean gray-scale ratio of Emp to β-actin protein (MGREAp) in groups without EPO treatment, or with EPO treatment for 30 and 90 days. These data are representative of three independent experiments. *n* = 3. *P < 0.05.

In order to explore the correlation of Emp and erythroblastic island formation in bone marrow in patients with severe anemia treated with EPO. Before EPO treatment, the erythroblastic islands were barely found in bone marrow smears. After EPO treatment, the number of erythroblastic islands was dramatically increased in the bone marrow smear. (Table 2, Figure 3A). The Emp protein was undetectable in mononuclear cells from bone marrow in patients not treated with EPO. However, after EPO treatment the Emp protein was significantly increased in mononuclear cells from bone marrow, in accordance with enhanced island formation (Figure 3B).

## Discussion

HSCT is a potentially curative therapy for a variety of hematological disorders and cancers of the blood or bone marrow, such as multiple myeloma or leukemia. Hematopoietic evaluation of the patients after HSCT is very important.

In humans, the functional unit for definitive erythropoiesis is the erythroblastic island, a multicellular structure composed of a central macrophage surrounded by developing erythroblasts. Erythroblast-macrophage interactions play a central role in the terminal maturation of erythroblasts [1,2,9]. Reconstitution and increase of erythroblastic islands in bone marrow was usually used as a marker for successful HSCT. Erythroblast-macrophage protein (Emp) was initially identified as a mediator of erythroblast-macrophage interactions during erythroid differentiation. The Emp protein [1,2,8,16,18] plays an important role in normal maturation process of erythroblastic cells in erythroblastic islands in bone marrow. More recent studies have shown that targeted disruption of Emp leads to abnormal erythropoiesis. Unfortunately, the involvement of Emp in HSCT remains uncertain. In this report, we showed that Emp protein levels were correlated with erythroblastic island formation in bone marrow.

Emp was expressed in a variety of hematopoietic (and indeed many non-hematopoietic) cells, including erythroblasts and macrophages [21]. However, Emp expression increased in erythroblasts when hematopoiesis took place in bone marrow (Table 3). Interestingly in this study, Emp mRNA was detected in all the groups, whereas the Emp protein was only detectable in bone marrow mononuclear cells in HSCT group (Figure 2C). These results suggested that Emp might be a potential marker for bone marrow hematopoietic evaluation.

EPO, a kind of hormone preventing erythroid progenitors’ apoptosis [22], had been widely used in clinics [22-24]. Erythroblastic islands were barely found in bone marrow smears before EPO therapy and were dramatically increased after EPO treatment (Table 2, Figure 3A). Meanwhile, Emp protein level was significantly increased in mononuclear cells from bone marrow in patients receiving EPO treatment (Figure 3B). The data indicate that Emp protein expression could have a close correlation with hematopoietic island formation in bone marrow.

Taken together, our findings provide that Emp protein expression in bone marrow has a close relation with hematopoietic island formation, which might be a potential marker for hematopoietic evaluation in clinic. Thus, our findings suggest that erythobalst island formation is likely an essential feature of erythropoiesis after HSCT. Future studies will be required to assess if Emp levels directly correlate with functional erthropoiesis in this model.

## Conclusions

The Emp expression correlates with erythroblastic island formation and has an important function for bone marrow hematopoiesis. Based on our data, we suggest that the Emp is a potential biomarker for hematopoietic evaluation of HSCT patients.

## Competing interests

The authors declare that they have no competing interests.

## Authors’ contributions

XM carried out the molecular genetic studies, statistical analysis, and drafted the manuscript. XS participated in the molecular genetic studies. FL carried out the immunoassays. GL performed the statistical analysis. LH conceived of the study, and participated in its design and coordination and helped to draft the manuscript. All authors read and approved the final manuscript.
